# Microarray analysis of retinal gene expression in chicks during imposed myopic defocus

**Published:** 2008-08-31

**Authors:** Ruth Schippert, Frank Schaeffel, Marita Pauline Feldkaemper

**Affiliations:** Institute for Ophthalmic Research, Section of Neurobiology of the Eye, University Eye Hospital Tuebingen, Tuebingen, Germany

## Abstract

**Purpose:**

The retina plays an important regulatory role in ocular growth. To screen for new retinal candidate genes that could be involved in the inhibition of ocular growth, we used chick microarrays to analyze the changes in retinal mRNA expression after myopic defocus was imposed by positive lens wear.

**Methods:**

Four male white leghorn chicks, aged nine days, wore +6.9D spectacle lenses over both eyes for 24 h. Four untreated age-matched male chicks from the same batch served as controls. The chicks were euthanized, and retinas from both eyes of each chick were pooled. RNA was isolated and labeled cRNA was prepared. These samples were hybridized to Affymetrix GeneChip Chicken Genome arrays with more than 28,000 characterized genes. After comparison of multiple normalization methods, GC-RMA and a false-discovery rate of 6% was chosen for normalization of the data. The expression of 16 candidate genes was further studied, using semiquantitative real-time RT–PCR. In addition, the expression of the mRNA of some of these candidate genes was assessed in chicks that wore either +6.9D lenses for 4 h or −7D lenses for 24 h.

**Results:**

123 transcripts were found to be differentially expressed (p<0.05; at least 1.5-fold change in expression level), with an absolute mean fold-change of 1.97±1.16 (mean±standard deviation). Nine of the sixteen genes that were examined by real-time RT–PCR were validated. Regardless of whether positive or negative lenses were worn, six of these nine genes were regulated in the same direction after 24 h: arginyltransferase 1 (*ATE1*), E74-like factor 1 (*ELF1*), growth factor receptor-bound protein 2 (*GRB2*), SHQ1 homolog (S. cerevisiae) (*SHQ1*), spectrin, beta, non-erythrocytic 1 (*SPTBN1*), prepro-urotensin II-related peptide (*pp-URP*). Three genes responded differently to positive and negative lens treatment after 24 h: ATP-binding cassette, sub-family C, member 10 (*ABCC10*), CD226 molecule (*CD226*) and oxysterol binding protein 2 (*OSBP2*).

**Conclusions:**

The validated genes that were regulated only by myopic defocus may represent elements in a pathway generating a “stop-signal” for eye growth. Some of the genes identified in this study have so far not been described in the retina. Further investigation of their function may improve the understanding of the signaling cascades in emmetropization. More general, published microarray data are variable among different animal models (mouse, chick, monkeys), tissues (retina, retina/retinal pigment epithelium), treatments (diffusers, lenses, lid-suture), as well as different treatment durations (hours, days), and comparisons remain difficult. That only a small number of common genes were found emphasizes the need for careful normalization of the experimental parameters.

## Introduction

The high incidence of myopia is a problem throughout the industrialized world, especially in Southeast Asia [1-3]. Although it is generally accepted that there is a major genetic influence, it has become clear that the visual experience is important as well [4,5]. Studies in several animal models (e.g., tree shrew, chick, monkey, guinea pig, mouse) have shown that manipulating the retinal image features can induce alterations in the rate of ocular growth [6-10]. Treatment with negative lenses (hyperopic defocus) or diffusers leads to myopia, while treatment with positive lenses (myopic defocus) induces the development of hyperopia. Experiments in which the optic nerve of deprived and lens-treated animals was sectioned revealed that an intact link between the retina and the brain is not necessary for the development of experimental myopia or hyperopia [11-13]. These experiments show that the retina controls refractive development by processing visual signals largely without the involvement of the brain. However, the biochemical pathways underlying these processes require further characterization.

Changes in retinal concentrations of several substances have been demonstrated to be associated with altered eye growth, including dopamine [14], glucagon [15-17], early growth response factor-1 (EGR1 or ZENK) [18-21], retinoic acid [22-24], vasoactive intestinal polypeptide [13,25], and others [26]. Recently, microarray studies provided further candidates in chicks, monkeys and mice [27-29]. A study performed by McGlinn et al. [27] on retina, retina/retinal pigment epithelium (RPE) tissue of form-deprived chicks revealed new genes such as bone morphogenetic protein 2, prepro-urotensin II-related peptide**(*pp-URP)* and mitogen-activated protein kinase phosphatase 2. The authors found that the changes in mRNA expression induced by form-deprivation were small, and that only a small number of genes showed any responses. Brand et al. [28] found significant changes in the mRNA concentration of *Egr1*, the Finkel-Biskis-Jinkins osteosarcoma oncogene (*Fos*), thymoma viral oncogene homolog 2, and others in response to the treatment of mice with diffusers. Tkatchenko et al. [29] observed that the mRNA expression of several genes associated with cell division were changed in primate retina following lid-fusion.

As yet, no distinct pathways for the retinal control of eye growth have been defined. Therefore there are still no validated targets for pharmacological intervention. The present study was aimed at finding new candidate genes that could be involved in the generation of a stop signal for axial eye growth. Unlike other studies, which addressed the signals for myopia development induced by negative lens or diffuser wear, this study was designed to identify genes involved in hyperopia development. To this end, both eyes of each chick were covered with positive lenses. Since hyperopia development requires an inhibitory signal for axial eye growth, identification of such a signal could provide an effective way to inhibit myopia. Retinal mRNA expression patterns were compared between positive lens-treated and untreated chicks. The visually induced changes in the transcription of potential candidate genes were further investigated by semiquantitative real-time RT–PCR.

## Methods

### Treatment of the animals

All experiments were conducted in accordance with the ARVO statement for the use of Animals in Ophthalmic and Vision Research and approved by the University Commission for Animal Welfare (reference AK 6/05). One day old male white leghorn chickens obtained from a local hatchery in Kirchberg, Germany, were raised under a 12 h:12 h light-dark cycle (light-onset: 8:00 AM and light-offset: 8:00 PM) with unrestricted access to water and food pellets (chicken breeding pellets, RKW-Sued, Würzburg, Germany). No additional vitamins or supplements were added. On the day before the experiment started, the chicks were placed under diethylether anesthesia and a velcro ring was glued to the feathers around each eye. At the age of 9 days, 18 chicks were split into three experimental groups of six. One group wore +6.9D lenses binocularly for 24 h. The second group wore +6.9D lenses for 4 h, while the third group wore −7D lenses for 24 h. For each group, six untreated control chicks of the same batches were included in the analyses to ensure that the treated samples and the control samples were similarly processed. Because of the short treatment period, no additional measurements of the chicks were performed (e.g., A-scan or measurement of refractive state). Also, we did not control for the viewing distances because it was already shown that chicks receive consistent myopic defocus on the retina under our experimental conditions [30]. Chicks were killed by an overdose of diethylether. Afterwards, both eyes were enucleated and the retinas were separated. The retina of both eyes were pooled and RNA was extracted using the RNeasy Mini Kit (Qiagen, Hilden, Germany), according to the manufacturer’s instruction. Microarray experiments were performed with four of the six samples of the group treated with +6.9D lenses for 24 h. All six samples of each group were analyzed using real-time RT–PCR.

### Microarrays

Microarray analysis was performed by the Affymetrix Resource Facility at the University of Tuebingen. RNA was quantified and checked for quality with the Agilent 2100 Bioanalyzer (Agilent Technologies, Palo Alto, CA). The RNA integrity numbers (RIN) ranged from 9.2 to 9.5 (with 1 being the most degraded profile and 10 being the most intact) [31]. The GeneChip Chicken Genome Array (Affymetrix, Santa Clara, CA) was used with a coverage of 32,773 transcripts, corresponding to over 28,000 chicken genes.

Next 1.5 µg total RNA (1.5 µg) was reverse transcribed using a T7-oligo (dT) promotor primer in the first-strand cDNA synthesis. After RNaseH-mediated second-strand synthesis, the double-stranded cDNA was purified and served as a template in the subsequent in vitro transcription reaction. This was performed in the presence of T7 RNA polymerase and a biotinylated nucleotide analog/ribonucleotide mix for complementary RNA (cRNA) amplification and biotin labeling. The biotinylated cRNA targets were cleaned up according to the standard affymetrix protocol, fragmented, and hybridized to GeneChip expression arrays, followed by an automated washing and staining protocol (with streptavidin phycoerythrin conjugate) on the fluidics station. Scanning and analysis were performed using the Affymetrix Microarray Suite Software (version 5.0).

The signal intensities were analyzed using ArrayAssist 4.0 (Stratagene, La Jolla, CA). Results were corrected for multiple testing using the Benjamini/Hochberg paradigm with a false discovery rate (FDR) of 6%. Each data set was normalized using three different normalization methods (GC-RMA, RMA, MAS5), with GC-RMA being our method choice for further analyses. The mRNA expression levels of genes of treated and untreated chicks were compared using unpaired *t*-tests.

### Real-time RT–PCR

Sixteen genes that were found in the microarray analysis were selected for further analysis. Selection criteria were high fold-changes or p-values, interesting biologic functions (e.g., transporter- or molecular transducer activity), differential expression of several probe-sets of one gene present on the chip and already described changes of mRNA expression of these genes in other microarray-studies. Semiquantitative real-time RT–PCR was used to assess mRNA expression levels of the selected genes. Next, 1 µg RNA from each sample was reverse transcribed with M-MLV reverse transcriptase (Promega, Mannheim, Germany) using 0.5 µg oligo(dT)15 primer and 50 ng of a random primer mixture (Invitrogen, Solingen, Germany). QuantiTect SYBR Green master mix kit (Qiagen) was used for fluorescence detection on the iCycler iQ Multicolor Real-Time PCR Detection System from Bio-Rad (Hercules, CA). Samples were analyzed in triplicate with a template amount corresponding to 2 ng of RNA. Hypoxanthine-phosphoribosyl-transferase (*HPRT*) was used as a housekeeping gene. Primer sequences and NCBI accession numbers are shown in Table 1, as well as the averaged relative expression levels of the control animals determined by microarray analysis. The amplification efficiency (E) for all gene-specific primer pairs were evaluated using a dilution series as previously described [32]. Briefly explained, it was calculated from cDNA standard curves by means of the slope of the regression line with the equation: Efficiency = [10(-1/slope)] - 1, whereby a value of 1.0 corresponds to 100% efficiency and a value lower than 1.0 indicates an inhibition of the reaction or poor primer binding.

**Table 1:** Description of primers

**Gene**	**NCBI accession number**	**Forward primer (5′-3′)**	**Reverse primer (5′-3′)**	**Amplicon**	**Averaged relative signal intensity of controls**
*HPRT*	NM_204848	TGGCGATGATGAACAAGGT	GCTACAATGTGGTGTCCTCCC	162 bp	735
*ABCC10*	XM_419506	CTATGCTCTCGGGCTCTTTG	GACAGTGAAGCAGGAAAGGC	166 bp	53
*ACVR1*	NM_204560	CGGAGGTCTTGGACGAAAC	GGATCATTTGGAACCAGGTC	166 bp	19
*ATE1*	NM_001079733	TACTGGCTGGATGGGAAGATAA	GCTTTCTCGTGAAGTTGC	164 bp	93
*CD226*	XM_001235284	TAGACAATGTGGAAGGAAGGT	TGTATGCCATAGATAGGATGC	169 bp	111
*CHRNB2*	NM_204813	TGCTGGTGACCTTCTCCATCGT	AGTTCTGCTGCGGCTGCTT	150 bp	35
*ELF1*	NM_001006269	CACAGGAACAAAGGGAGGAT	GGATGTACTGGCTGCGTAGA	153 bp	13
*ETV5*	XM_422651	TCTGGCAGTTCCTCGTCA	GCCCTTCTCGTAGTAGTAGCG	191 bp	287
*GHRHR*	NM_001037834	CTTGGCATTCGGCTTTATTT	GGCACAGTCCATCTTGTCCT	170 bp	7
*GNAT2*	NM_204690	GCTCCACATCACTGTTCTGCTG	TGCCCGTTTCCTCTTCCCCT	224 bp	10078
*GRB2*	NM_204411	ATCTCCTCTGGGTGGTGAAG	GATAAAGTCTCCACGGCGG	212 bp	73
*MKP2*	NM_204838	AGCCCTGCTGAACGTCTCA	AGGGATGCACTTGTACTGGTAGTG	70 bp	29
*OSBP2*	XM_415293	GTGGTGAGTGATGCTGATGG	CTTTGGGGACAGTGTCTGGT	149 bp	14
*REEP6*	XM_424848	TGGTGTATGGCGTCTTCAGT	CACGGTGGTGTTTGAGGAA	180 bp	167
*SHQ1*	XM_414429	CGAAGAAATCAAGGACAGCA	CAAATCCATAGTAGCACTGAAG	159 bp	28
*SPTBN1*	XM_419291	GCCATTGAAACAGACATTG	CCCACAGGCGTATAACATTG	136 bp	20
*pp-URP*	NM_206989	TGTGAAGCCTCAGCACCCTCT	CCATCCTCCCCCAAACCTACT	148 bp	159

Shown are all genes that were investigated by real-time RT–PCR, together with NCBI accession number, primer sequence and product length. The averaged relative expression level of the control animals was determined by microarray analysis and reflects the relative abundance of the transcript.

### Pathway analysis

GC-RMA normalized microarray data was analyzed using the software Ingenuity Pathways Analysis (Ingenuity Systems). As the program did not contain annotations for the chicken genome, the human homologs of the 123 differentially expressed genes were used instead.

### Statistics and data analysis

Data were analyzed using Excel and the software package JMP 5.1 (SAS Institute GmbH, Munich, Germany). The mean cycle threshold (Ct) value of each triplet was taken and transformed into Mean Normalized Expression (MNE), with *HPRT* as a housekeeping gene as previously described [16]. Data were transformed with the common logarithm to obtain a more normal distribution. Since real-time RT–PCR of each treatment group (+6.9D/24 h, +6.9D/4 h, −7D/24 h) was performed together with an individual control group, we used an unpaired t-test to compute the mean differences (deltaMNE) and the 95% confidence interval in the MNE-values between every treated group and its individual control group. Given that there was no change in any of the genes tested after 4 h of +6.9D lens treatment, these data were not included in the subsequent analysis.

## Results

### Microarray analysis

Using a p-value that was below 0.05, a fold change (FC) of at least 1.5-fold and a false-discovery rate of 6%, we found 123 genes to be differentially expressed after 24 h of treatment with +6.9D lenses (Appendix 1, GC-RMA normalized microarray data). Interestingly, two already known candidate genes, glucagon and *ZENK *(*EGR1*), were part of this list. The comparison of mRNA levels in retina of eyes treated with positive lenses to those without lenses revealed maximum fold changes of +11.8 (upregulation) of the ras homolog gene family, member G (*RHOG*) mRNA and −7.7 (downregulation) of *CD226* mRNA (Appendix 1). We found 67 of all differentially expressed genes were upregulated in the positive lens-treated eyes, and 56 were downregulated (unpaired *t*-tests). According to gene ontology annotations, most of those with known function had catalytic activity or were involved in binding (Appendix 1).

The cytobands for the known human counterparts are given in Appendix 1 as well. The human homologs of six genes were found to be localized in chromosomal regions that are already known to be associated with myopia. These genes are shown in italic font and are underlined. Tetratricopeptide repeat containing 3 (*TMTC3*) is located on the MYP3 locus, similar to EMO2 (*LOC416957*) on MYP6, glutamine and serine rich 1 (*QSER1*) on MYP7, neuroligin 1 (*NLGN1*) on MYP8, doublecortex (*DCX*) on MYP13, and grainyhead-like 3 (*GRHL3*) on MYP14. Until now, none of these genes were considered as a candidate gene for human myopia.

Different genes were identified if other normalization methods were used. The MAS5 normalization method yielded a high number of differentially expressed genes (1030) whereas RMA normalization yielded only 31 differentially regulated genes (cut-off level was again a minimum FC of 1.5 and a p-value below 0.05 in both cases). In a comparison of all normalization methods, we observed that only 21 genes appeared in all three lists.

Data obtained by GC-RMA normalization (123 genes) were also analyzed using Ingenuity Pathways Analysis software, but no distinct pathways were identified based on the changes in mRNA expression.

### Real-time RT–PCR

Sixteen genes were chosen for validation using real-time RT-RCR. Seven of these 16 genes were taken from the list of 21 that were found with all three normalization methods (*CD226*, *GHRHR*, *GNAT2*, *OSBP2*, *SHQ1*, *SPTBN1*, *pp-URP*), and nine additional genes were chosen from the list of differentially expressed genes that were obtained after GC-RMA normalization (genes shown in bold in Appendix 1). Table 2 compares the results of the real-time RT–PCR with the microarray analysis. The tissue originated from the chicks treated with +6.9D lenses for 24 h. All four microarray samples were tested by real-time PCR as well. In addition, two samples that were not subjected to microarray analysis were tested with real-time PCR.

**Table 2:** Results obtained by microarray analysis and real-time RT-PCR

**Gene**	**Fold change PCR**	**p-value PCR**	**Fold change MA**	**p-value MA**
***ABCC10***	**-1.23**	**0.0385**	**-1.59**	**0.0047**
*ACVR1*	1.17	0.1975	-1.63	0.0180
***ATE1***	** 1.32**	**0.0149**	** 1.56**	**0.0093**
***CD226***	**-2.54**	**0.0318**	**-7.70**	**0.0247**
*CHRNB2*	1.31	0.0082	-1.50	0.0314
***ELF1***	** 1.24**	**0.0446**	** 1.83**	**0.0069**
*ETV5*	1.10	0.2014	-1.51	0.0000
*GHRHR*	2.07	0.1160	3.08	0.0056
*GNAT2*	1.09	0.4197	-2.49	0.0005
***GRB2***	** 1.27**	**0.0245**	** 1.50**	**0.0150**
*MKP2*	-1.36	0.1374	-1.75	0.0162
***OSBP2***	** 1.27**	**0.0059**	** 4.00**	**0.0412**
***pp-URP***	**-1.81**	**0.0045**	**-2.50**	**0.0010**
*REEP6*	1.23	0.0912	-2.03	0.0331
***SHQ1***	** 3.62**	**0.0077**	** 4.03**	**0.0214**
***SPTBN1***	** 1.39**	**0.0071**	** 2.90**	**0.0011**

Shown are data from retinal samples of chicks that were treated with +6.9D lenses for 24 h. The numbers represent fold-changes and p-values for the changes of the investigated genes in both the real-time RT-PCR (PCR) and the microarray (MA) experiment. Genes shown in bold were further tested with real-time RT–PCR in two other experimental paradigms: treatment with +6.9D lenses for 4 h or with −7D lenses for 24 h.

Nine out of the 16 tested genes could be confirmed by real-time PCR and are shown in bold font in Table 2. We compared the fold-changes in gene expression levels between GC-RMA microarray scores and real-time PCR of all tested genes and found a significant orthogonal correlation (correlation coefficient=0.759; n=16).

**Figure 1:**
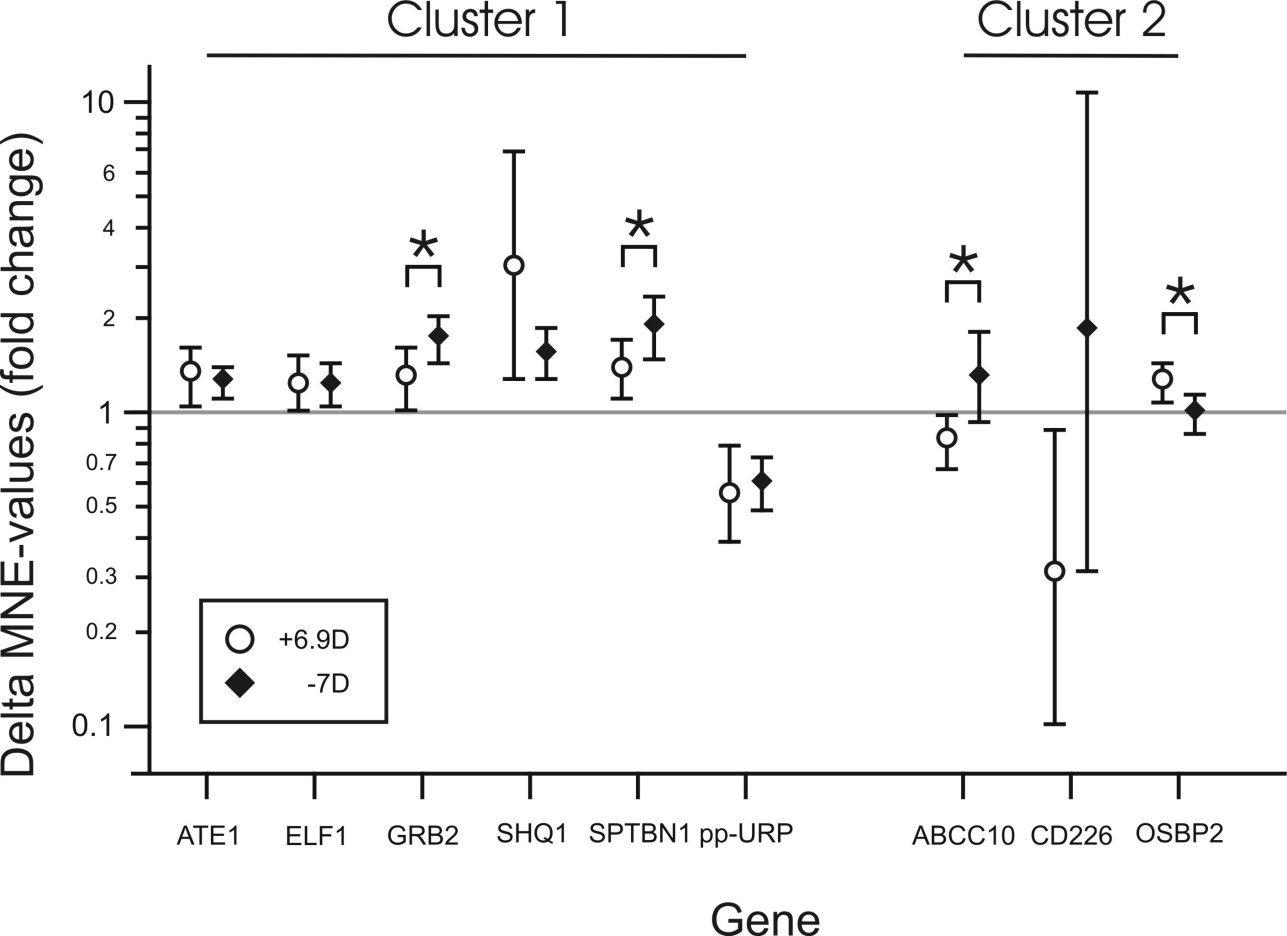
Results of the real-time PCR experiment. The mean differences in gene expression (shown as delta Mean Normalized Expression values, deltaMNE) and their 95% confidence intervals between the lens-treated groups (24 h treatment with +6.9D and −7D lenses, respectively) and the untreated control groups (n=6 animals each) are shown for the nine genes for which the microarray data could be confirmed. The horizontal gray line at fold change 1 indicates no change. Asterisks denote significant differences (p<0.05) between the two groups. Unpaired *t*-tests were performed, and not corrected for multiple testing. Genes were assigned to clusters depending on the directions of the changes (Cluster 1: changes in the same direction under both conditions; Cluster 2: only regulated in response to positive lens treatment).

Two additional experimental paradigms were used to further elucidate the possible roles of the nine validated genes (+6.9D lenses for 4 h, −7D lenses for 24 h). None of the nine genes showed significantly altered mRNA expression levels after 4 h of treatment with +6.9D lenses (n=6; results not shown). Changes in expression following either −7D or +6.9D lens treatment (n=6 for each group) for 24 h are shown in Figure 1. The mean differences of the normalized gene expressions (deltaMNE) between each treated group and its individual control group, as well as the 95% confidence intervals are plotted.

The horizontal gray line in Figure 1 represents the level of no difference in expression. No overlap between the 95% confidence interval bars and this line indicates that there was a statistically significant difference between the treated group, and the respective untreated control group. Significant differences between negative and positive lens-treated groups are denoted by asterisks in Figure 1 (p<0.05 each).

Based on the real-time RT–PCR experiment, the genes could be clustered into two categories. The first cluster includes genes that showed changes in the same directions, no matter whether positive or negative lenses were used: “image sharpness detection” (*ATE1*, *ELF1*, *GRB2*, *SHQ1*, *SPTBN1,* and *pp-URP*). In this cluster, positive lenses as well as negative lenses induced significant changes. The second group includes those genes that were regulated only in response to positive lens wear, but remained unchanged in response to treatment with negative lenses: “sign of defocus detection” (*ABCC10*, *CD226*, *OSBP2*).

*GRB2*, *SPTBN1*, *ABCC10,* and *OPBP2* were differentially expressed in response to +6.9D and −7D treatment. Both *GRB2* and *SPTBN1* showed an upregulation after positive and negative lens treatment but the upregulation was more pronounced in the negative lens-treated animals. This suggests a graduated regulation of the transcription of these genes with the absolute amount of defocus. *ABCC10* expression was significantly downregulated in the positive lens-treated animals, but upregulated in the negative lens-treated animals. *OSBP2* displayed no changes in response to the treatment with negative lenses, but there was a significant upregulation in the positive lens-treated animals.

The data discussed in this publication have been deposited in the National Center for Biotechnology Information (NCBI's) Gene Expression Omnibus (GEO) and are accessible through GEO Series accession number GSE11439.

## Discussion

While changes in retinal gene expression associated with the development of myopia have been described in previous reports, this is the first study to examine gene expression changes during development of experimental hyperopia. This approach was chosen because we were interested in potential candidates for “stop signals” of axial eye growth. Using GC-RMA for normalization of the microarray data, we found 123 differentially regulated genes after one day of positive lens wear. The finding that two already known candidate genes, glucagon and *ZENK* (*EGR1*), were in this list underlines the usefulness of the microarray technique in discovering genes that underlie the targeted biologic processes. Expression changes were confirmed by real-time PCR for nine of 16 genes. Changes in mRNA expression patterns of the validated genes were further studied in additional treatment groups. A short treatment period of 4 h did not influence the mRNA expression level of these genes, whereas some of them showed significant changes after one day of negative lens treatment.

### Microarray data

#### Different normalization methods

It was not especially surprising to see that the different normalization methods (GC-RMA, RMA, and MAS5) produced variable results regarding the total number of differentially expressed genes, with little overlap between the studies (only 21 genes). This was similar to a previous microarray study undertaken by Brand et al. [28]. There are major differences between GC-RMA, RMA, and MAS5 normalization methods: MAS5 corrects for hybridization to the mismatch probes for that particular probe set, whereas GC-RMA and RMA alternatively calculate a background adjustment step that ignores the mismatch intensities [33]. Millenaar et al. [34] evaluated multiple normalization methods and found MAS5 to have the most distinct outcome compared to the other two procedures. However, the differences were much smaller in their study compared to ours. We chose seven genes that were represented in all lists (GC-RMA, RMA, and MAS5) for validation by semiquantitative real-time RT–PCR. As a result, five genes could be verified—which represents a higher percentage than with the GC-RMA normalized data list. We therefore agree with the conclusion by Brand and colleagues [28] that each normalization method likely provides only a fragmentary picture of all changes in gene expression.

#### Magnitude of changes in gene expression

We found only small changes in gene expression in positive lens-treated chicks, involving 123 genes with an average fold change of ±1.96. The magnitudes of changes are in line with the findings of other microarray studies [27,28]. It is known that even small changes in the expression of biologically relevant transmitters or neuromodulators can cause large effects. For instance, the drop in retinal dopamine levels associated with the development of form-deprivation myopia in chicks (e.g., Stone et al. [14]) did not exceed 30%. In addition to the regulation at the transcriptional level, the processing/transport and the translation and stability of mRNA regulate the protein (expression) level. For instance, a study in yeast, where changes in mRNA levels and protein levels were compared, showed that at least 20% of the changes in mRNA concentrations did not show up as parallel changes in protein levels [35]. And finally, as the total retinal tissue was processed, potentially large local cell-specific changes in mRNA concentrations could have been averaged out.

#### Treatment time and biochemical changes

It has been found in previous studies [36,37] that changes in retinal mRNA levels occur as early as after 24 h of treatment with spectacle lenses. Negative lens treatment was observed to cause a significant decline in glucagon mRNA levels [16] and positive lens treatment an increase in glucagon mRNA levels [38]. The same treatment duration induced changes in proteoglycan synthesis in the chick sclera [39] and changes in expression of collagen-binding integrin receptors in tree shrew sclera [40], suggesting that active remodeling of the distinct layers of the eye was already in progress.

Some changes in gene expression can also occur much earlier. For example, the concentration of the mRNA of the transcription factor *ZENK* is changed already after 15–30 min [18-20]. Some of the early events—for example, *ZENK* expression changes—may persist after one day of lens treatment (*ZENK* mRNA level remains low after one day of negative lens treatment) or even reverse the direction of changes (*ZENK* mRNA levels are upregulated by short periods of positive lens wear [19] but seem to be downregulated by longer periods of positive lens treatment [41]).

That the nine validated genes did not show any significant changes, or even a trend toward a change, after only 4 h of positive lens treatment suggests that they are not essential in the early signaling cascade in the retina following imposed defocus.

### Validation and characterization of genes with real-time RT–PCR

It was possible to confirm nine out of 16 differentially expressed genes. Nevertheless, some of the microarray results were not confirmed. One explanation for the failure of the validation of some of the microarray results may be alternative transcript usage as a technical and conceptual issue in comparing across species and studies. A recent study characterized alternate splicing and tissue-specific expression in the chicken from expressed sequence tags [42]. The authors suggested that alternate splicing may occur in 50%–60% of the chicken gene set with an average of more than two transcripts per gene which undergo this process. This underlines that real-time PCR validation may fail in some cases, because most genes have several transcripts: the sequences that were amplified with the primer pairs of three of the genes that could not be validated in our study (*REEP6, MKP2*, and* GHRHR*) did not include the same region of the gene against which the microarray probes had been designed. The other four genes that could not be validated were presented more than once on the microarray, with only one probe set showing differential expression. The possibility of the presence of yet to be known isoforms can therefore not be excluded. Moreover, microarrays and semiquantitative PCR require and utilize vastly different normalization methods.

### Comparisons with other microarray studies

In similar microarray studies [27-29], only a small number of genes were changed by visual conditions that induce refractive errors. Unfortunately, there is little overlap among the lists from different studies. Different normalization methods may account for part of the problem, but differences in treatment paradigms, animals, and samples (pure retina versus retina/RPE) are also the case. Comparisons of different studies are shown in Table 3, together with the individual normalization methods.

**Table 3:** A comparison of gene lists with other studies

**Affymetrix ID**	**Gene symbol**	**FC Schippert**	**FC other**s	**p value Schippert**	**Gene title**	**Normalization**
Comparison with McGlinn et al. [27]						
Gga.19434.1.S1_at		-1.64	1.26	0.0252	Finished cDNA, clone ChEST955o8	both RMA
Gga.8944.3.S1_s_at	*LOC424393*	1.78	1.23	0.0004	similar to KIAA1096 protein	both RMA
Gga.9482.1.S1_at	*LOC404534*	-1.83	-1.67	0.0004	prepro-urotensin II-related peptide	both RMA
Comparison with Brand et al. [28]						
GgaAff × 0.21017.1.S1_s_at	*CALD1*	-1.65	-2.65	0.0295	caldesmon 1	both MAS5
Gga.10521.1.S1_s_at	*GARNL1*	1.57	1.51	0.0016	GTPase activating Rap/RanGAP domain-like 1	both MAS5
Gga.9350.1.S1_s_at	*KNTC2*	-4.41	-3.52	0.0491	kinetochore associated 2	both MAS5
Comparison with Tkatchenko et al. [29]						
GgaAff × 0.4150.3.S1_s_at	*ARHGEF12*	1.55	Upregulation (FC unknown)	0.0213	similar to Rho guanine nucleotide exchange factor 12	GCRMA/GAPDH

Comparisons of the list of differentially expressed genes found in the present study with microarray studies from other groups [27-29]. Shown are Affymetrix ID, gene symbol, fold changes of the respective gene in the present study (FC Schippert) and in the respective other studies (FC others), p-values obtained in this study (p-value Schippert), gene title, and normalization methods used.

McGlinn et al. [27] studied form vision-deprived chicks after 6 h and 3 days and analyzed the retina/RPE tissue with the GeneChip Chicken Genome Arrays (Affymetrix). Three genes were significantly changed in both their study and ours: *pp-URP*, *LOC424393* (the homolog to the human BAT2 domain containing 1), and the clone *ChEST955o8*. Not much is known about the function of pp-URP (see McGlinn et al. [27] for more details). As stated by these authors, pp-URP merits future investigation since it is implicated in the activation of the urotensin receptor, which then is able to stimulate growth signaling pathways [43]. Since *LOC424393* and *pp-URP* were changed in the same direction during form deprivation myopia and lens-induced hyperopia development, there is no link to the sign of axial eye growth changes. Unfortunately, no information is available about the only gene that was differentially regulated during form-deprivation myopia and lens-induced myopia (*ChEST955o8*).

Brand et al. [28] deprived mice of form vision in one eye for different durations (30 min, 4 h, 24 h). They then compared the mRNA expression in the form-deprived retina to the one in the fellow eye, which had been treated with neutral density filters to match light attenuation. Three genes were consistently changed in both their study and ours. Caldesmon 1 mRNA, an ubiquitous actin- and calmodulin-binding protein [44], which is also a substrate for mitogen-activated protein kinase [45] and other serine and threonine kinases [46-48] was downregulated in both studies, GTPase activating Rap/RanGAP domain-like 1 mRNA was upregulated in both studies, and kinetochore associated 2 mRNA was downregulated in both studies.

Finally, Tkatchenko et al. [29] performed a microarray study of retinas from rhesus macaques and green monkeys who had been deprived of form vision by surgical lid-fusion. A comparison between both lists is difficult because the authors had constructed their own microarrays and had normalized the data against the expression of glyceraldehyde-3-phosphate dehydrogenase mRNA. Therefore, the GC-RMA normalized data were compared with the list of Tkatchenko and colleagues. Only one gene showed up in both lists: Rho guanine nucleotide exchange factor 12 mRNA, which was upregulated in both cases.

Since all these genes, except for *ChEST955o8*, were regulated in the same direction both during myopia and hyperopia development, they are most likely not linked to pathways specific for either stimulation or inhibition of eye growth.

### Localization of genes to known myopia loci

Human homologs, if known, were tested for possible localization at already known chromosomal susceptibility loci. Six genes mapped to regions that were already known to be associated with myopia in different families. Five of them were upregulated during induction of hyperopia with positive lenses (*DCX*, *NLGN1*, *QSER1*, *TMTC3,* and *LOC41695*) and one was downregulated (*GRHL3*). DCX is a cytoplasmatic protein suspected to direct neuronal migration by regulating the organization and stability of microtubules in the developing cortex [49]. NLGN1 is a neuronal cell-surface protein that may be involved in the formation and remodeling of central nervous system synapses [50]. No further information is available at present about the possible function of QSER1, TMTC3, and C22orf30 (the human homolog of LOC416957). GRHL3 probably acts as a transcription factor during development [51]. Although none has been detected in human myopia linkage studies, these genes may represent new candidates for future linkage analyses.

### New candidate genes

The genes that were closer investigated by real-time RT–PCR are described in more detail in the next section. None of these genes has a known function in the retina.

#### ABCC10

ABCC10 is supposed to be a lipophilic anion transporter, most likely involved in phase III (cellular extrusion) of detoxification [52]. So far, no involvement in retinal processing has been proposed. Therefore, speculations about the function of ABCC10 in the signaling cascade in the retina are difficult. Nevertheless, ABCC10 merits further investigation because it was downregulated in positive lens-treated chicks and, compared to this group, upregulated in negative lens-treated animals.

#### ATE1

This protein is an enzyme that is involved in the targeting of proteins for ubiquitin-dependent degradation [53]. It has been shown that in ATE1^−/−^ embryos, the G_q_/G_i_-activated extracellular signal-regulated kinase pathways were impaired. In these embryos, the mRNA expression of v-jun sarcoma virus 17 oncogene homolog, *FOS*, 3-phosphoinositide dependent protein kinase-1 and Cyclin D1 was found to be downregulated by roughly twofold [54]. Despite the upregulation of *ATE1* in both the positive- and the negative lens-treated group, none of the aforementioned genes was found to be differentially expressed in our study.

#### CD226

This glycoprotein is expressed on the surface of natural killer cells, platelets, monocytes, and a subset of T cells. It is a member of the immunoglobulin superfamily and mediates cellular adhesion to other cells bearing an unidentified ligand. Cross-linking CD226 with antibodies initiates platelet activation and aggregation in a process dependent on the Fc receptor and protein kinase C activation [55,56]. That *CD226* was most strongly downregulated in the 24 h positive lens-treated animals lends credence to the belief that these changes are somehow related to changes in axial growth—even if the mode of action remains unknown.

#### ELF1

This transcription factor regulates, among others, inducible gene expression during T cell activation [57]. ELF1 and repellent axon guidance signal have been implicated in the control and development of the retinotectal projection [58]. *ELF1* was upregulated after positive and negative lens treatment, suggesting that it is part of a more general response of the retina, rather than a specific signal for directional growth changes.

#### GRB2

GRB2 is an adaptor protein involved in signal transduction [59] by mediating the activation of RAS in complex with epidermal growth factor receptor and son of sevenless (SOS) [60]. It is implicated in the insulin pathway, and insulin itself has been shown to lead to excessive eye growth [61,62]. Insulin receptor substrate protein 1 is phosphorylated by the insulin receptor and is then able to bind GRB2, which then activates the mitogen-activated protein kinase pathway through its interaction with SOS. Additionally, GRB2 has been linked to the internalization of beta-adrenergic receptors in response to insulin [63].

There are 43 insulin-related, seven SOS-related, and 25 epidermal growth factor-related sequences on the Affymetrix chip, none of which was differentially expressed in the retina of positive lens-treated eyes. GRB2 interacts with 190 other proteins (see Human Protein Reference Database) and may well have other yet to be defined functions as suggested by the upregulation in negative lens-treated eyes as compared to the positive lens-treated eyes.

#### OSBP2

OSBP2 and OSBP1 have previously been shown to be located in the retina with OSBP1 being more abundant. *OSBP1* is not on the Affymetrix chip. OSBP1 and OSBP2 are expressed in different types of retinal cells with OSBP2 likely to be associated with membranes in a yet unknown way [64]. Oxysterols are oxidized byproducts of cholesterol that can cause cytotoxic effects, with low-density lipoprotein (LDL) being one of the major sources of oxidized cholesterol. OSBPs bind oxysterol, and are highly expressed in the RPE, which also expresses the LDL-receptor [65]. This suggests that the RPE has a mechanism to bind these oxysterols as they are released from the LDL complex. Another link could be established to apolipoprotein A1 (ApoA-I), whose level was elevated in the retina after the development of hyperopia [66], and the LDL-receptor. ApoA-I acts as a ligand for these receptors in chickens, comparable to the LDL receptor-related protein, which has been shown to regulate plasminogen and matrix metalloproteinase activation [67].

#### SHQ1

SHQ1 is an essential nuclear protein that is involved in rRNA processing pathways. Together with the protein NAF1 it is important in the initial steps of the biogenesis of small nucleolar RNAs (snoRNAs). SnoRNAs later form small nucleolar ribonucleoprotein particles, which are essential cofactors in ribosomal RNA metabolism. *SHQ1* was upregulated in both treatment groups, with a much higher variation between the six samples measured in the positive lens-treated group.

#### SPTBN1

SPTBN1 is a cytoskeletal protein involved with organizing receptor domains and possibly the control of vesicle traffic at the plasma membrane [68]. It interacts with calmodulin and calcium-dependent protease 1. SPTBN1 has been proposed to play a role in local mechanisms that can control rapid changes in membrane topography and skeletal organization and yet provide permanence and stability to the membrane between cycles of change [69]. Since *SPTBN1* mRNA expression was generally upregulated in both the positive- and the negative lens-treated group, we propose a more general role of this protein in retinal processing.

#### pp-URP

pp-URP II-related peptide is the precursor of the urotensin II paralog (URP). It binds to the G-protein coupled urotensin II receptor (UTS2R), which was also downregulated in our experiment (Appendix 1, Molecular Transducer Activity, FC=-1.51; Affymetrix ID: GgaAff × 0.1067.1.S1_at). If the effect of URP is indeed the same as of urotensin II, binding to the UTS2R leads to Gq protein activation, associated with activation of protein kinase C, protein tyrosine kinases, calmodulin, and phospholipase C [70-72]. Urotensin II also induces c-fos, which has been shown to be reduced in the retina of form-deprived mice [28]. This seems to support the possibility that pp-URP is involved in eye growth regulation. Nevertheless, the down-regulation of pp-URP in both cases (positive- and negative lens treatment) leads us to the assumption that this molecule is part of a general signaling pathway in the retina rather than a start- or a stop-signal for axial growth.

### Outlook

The current study merged in a long list of yet unknown genes that were regulated in the retina of chicks by exposure to myopic defocus. At present, no defined pathways could be associated with the observed changes. Apparently, the retina responds to treatment with positive lenses for 24 h with changes in several major signaling pathways (protein kinase C, G-protein-coupled receptors, mitogen-activated protein kinase). Comparisons with other published microarray studies remain inherently difficult because of differences in treatment protocols, animal models, and normalization methods. More closely matched experimental variables would help to improve the situation in the future, but it could also be that just more studies, especially studies that follow mRNA changes over time, would be sufficient to generate to a more coherent picture.

## Appendix 1. List of genes that were obtained by unpaired *t*-test and GC-RMA analysis

To access the data, click Appendix 1. This will initiate the download of a pdf that contains the file.

Chicks were treated for 24 h with +6.9D lenses. Eyes of untreated chicks of the same batches served as controls (4 individuals for each group). Affymetrix ID, Fold Change (FC), gene title and chromosomal position of the human homologs are shown. Genes were sorted after GO annotations. Genes that were localized in chromosomal loci of the human genome which are known to be associated with myopia are underlined and in italics. Genes that were chosen for real-time PCR validation are shown in bold.
